# Composition of the Gut Microbiota in Attention Deficit Hyperactivity Disorder: A Systematic Review and Meta-Analysis

**DOI:** 10.3389/fendo.2022.838941

**Published:** 2022-03-18

**Authors:** Ning Wang, Xuping Gao, Zifeng Zhang, Li Yang

**Affiliations:** ^1^ Department of Child and Adolescent Psychiatry, National Clinical Research Center for Mental Disorders and NHC Key Laboratory of Mental Health (Peking University Sixth Hospital), Peking University Sixth Hospital (Institute of Mental Health), Beijing, China; ^2^ Department of Psychiatry, Yan’an Third People’s Hospital, Yan’an, China

**Keywords:** attention-deficit/hyperactivity disorder, gut microbiota, dysbiosis, *Blautia*, systematic review and meta-analysis

## Abstract

**Background:**

The latest research accumulates information to explore the correlation between gut microbiota and neurodevelopmental disorders, which may lead to new approaches to treat diseases such as attention deficit/hyperactivity disorder (ADHD). However, the conclusions of previous studies are not completely consistent. The objective of the systematic review and meta-analysis was to identify evidence on the dysbiosis of gut microbiota in ADHD and find potential distinctive traits compared to healthy controls.

**Methods:**

Electronic databases, including PubMed, Embase, Web of Science, Cochrane Library, and PsycINFO, were searched up to August 24, 2021, using predetermined terms. Meta-analysis was performed to estimate the comparison of microbiota profiles (alpha and beta diversity) and the relative abundance of gut microbiota in ADHD patients and healthy controls.

**Results:**

A total of eight studies were included in the meta-analysis, containing 316 ADHD patients and 359 healthy controls. There was a higher Shannon index in ADHD patients than in healthy controls (SMD = 0.97; 95% CI, 0.13 to 1.82; *P* = 0.02; *I^2^* = 96%), but the significance vanished after sensitivity analysis because of high heterogeneity. No significant differences in other alpha diversity indexes were found. Regarding the relative abundance of gut microbiota, the genus *Blautia* was significantly elevated in ADHD patients compared with controls (SMD = 0.34; 95% CI, 0.06 to 0.63; *P* = 0.02; *I^2^* = 0%).

**Conclusions:**

Patients with ADHD had gut microbiome alterations compared to healthy controls. Though more studies with strict methodology are warranted due to the high heterogeneity, further studies to translate the findings of gut microbiota dysbiosis to clinical application in ADHD patients are needed and may guide targeted therapies.

**Systematic Review Registration:**

[https://www.crd.york.ac.uk/PROSPERO/display_record.php?RecordID=273993], identifier PROSPERO (CRD42021273993).

## Introduction

ADHD is one of the most common neurodevelopmental disorders and onset in early childhood, with a prevalence of 5.9% worldwide (1). It is a clinically heterogeneous disease that manifests with different combinations of symptoms, including inattention, hyperactivity, impulsivity, cognitive impairment, and imposes huge burdens on patients and families. The etiologies of ADHD are multifactorial, including genetic (2) and environmental (3) components.

ADHD patients usually have gastrointestinal symptoms (4) such as constipation, abdominal pain, fecal incontinence, accompanied by picky eating (5), and many other diseases (4–6) such as food allergies, asthma, and eczema. All these symptoms have been documented to be influenced by gut microbiota. Possible mechanisms involved microbial metabolites, amino acid metabolites, immune factors, and neurotransmitters (7).

Currently, the major therapeutic interventions for ADHD are medications, behavioral therapy, and cognitive training. While the efficacy of stimulant medications is validated by powered clinical trials, side effects, including decreased appetite, slight sleep delay, and cardiovascular risks, remain a cause for concern. In recent years, researchers have emphasized the importance of environmental factors such as the gut microbiota to investigate novel therapeutic approaches, including probiotics and prebiotics.

To date, several systematic reviews have shown the correlation between ADHD and gut microbiota, but no meta-analysis has been conducted. Thus, we performed this systematic review and meta-analysis to investigate the relationship between ADHD and gut microbiota and find potential distinctive traits in ADHD.

## Materials and Methods

### Protocol and Registration

The study was registered in PROSPERO (CRD42021273993) and strictly followed the PRISMA guidelines (8).

### Study Eligibility Criteria

Studies were included based on the following PICOS criteria.

#### Participants

Participants with confirmed ADHD were selected for the review, irrespective of age, gender, race, the existence of co-morbidities, and the use of medication. Animal studies were excluded in the review.

#### Interventions, Exposure(s)

No specific exposure was required. We were not interested in interventional studies.

#### Comparators

Comparator group was healthy controls (HCs) without ADHD diagnosis.

#### Outcomes

Studies were eligible if they report the differences between ADHD patients and HCs in gut microbiota diversity indices (alpha diversity and beta diversity) and relative or absolute abundance of microbial taxa.

#### Study Design

Studies were included if they were observational studies or controlled trials. Studies were excluded if they met any of the following criteria: case reports, conference presentations, reviews, expert opinions, or study protocol.

### Search Strategy

The most commonly used databases, including PubMed, Embase, Web of Science, Cochrane Library, and PsycINFO, were searched up to August 24, 2021, using the predetermined terms. The search strategy used is available in **Supplementary Material 1**. We did not set restrictions on language, year, or geographical location. Moreover, we manually searched the reference lists of identified articles to find potentially relevant studies and searched the System for Information on Grey Literature in Europe (SEGLE) and WorldCat for grey literature.

Two individual reviewers (NW, XPG) screened the titles and abstracts independently for possible articles. If there was an agreement between the two reviewers regarding a particular study, it was selected for further analysis; however, if there was disagreement, a third reviewer (LY) would determine whether the study qualifies for inclusion. The full texts of these potentially eligible studies were independently evaluated for eligibility by three reviewers (NW, XPG, ZFZ). Any disagreement between them was resolved by discussion or by a third reviewer (LY) when required.

### Data Extraction

If studies met the criteria mentioned above, then the data were extracted by one independent reviewer (NW) using a standardized extraction form. The second author (LY) will review all the extracted data with the team to resolve disputes, and the group (NW, XPG, ZFZ, LY) will finalize the data.

For all eligible studies, the following information was extracted: first author; year of publication; country; number, age and sex of ADHD patients as well as healthy controls; definition of ADHD; alpha diversity (microbial diversity within the same group’s samples, including observed operational taxonomic units (OTUs), observed species, Shannon diversity, Chao1 diversity, Simpson diversity); beta diversity (community diversity between different groups’ samples, including weighted UniFrac distances, unweighted UniFrac distances, Bray–Curtis distance, Jaccard distance); data on microbiota (including the phyla, order, family, genera, and species of microbiota detected and the methodology used for the microbiology assessment); dietary assessment; probiotics usage assessment.

### Quality Assessment

The quality of eligible studies was assessed using the Newcastle–Ottawa Quality Assessment Scale (NOS) (9) and evaluated by two reviewers (NW, XPG). The NOS assessed the quality of studies based on selection, comparability, and exposure, with a total score ranging from 0 to 9. A study of greater than 7 points is defined as a high-quality study.

### Data Synthesis

Different studies have investigated the gut microbiota’s taxonomic composition at different levels, such as phylum, order, family, genus and species, with a large number and limited overlap of findings. We excluded results if they were reported only in one study.

### Data Analysis

Studies included in this meta-analysis reported the comparison of gut microbiota between ADHD patients and controls, including alpha diversity and the relative abundance of bacteria of different phyla, families, and genera. These data were extracted from texts, figures, and supplementary materials. If only figures were given, we used Webplot-digitizer software (https://automeris.io/WebPlotDigitizer/) to extract these parameters from the graphs. Most data are expressed as the means ± standard deviations, and the others are presented as medians and interquartile ranges. We standardized all the data into the form of means ± standard deviations for subsequent analyses using a web-based tool (https://www.math.hkbu.edu.hk/~tongt/papers/median2mean.html).

This meta-analysis was undertaken using Review Manager 5.4 software. Data of gut microbiota were expressed as standardized mean difference (SMD). Heterogeneity was measured using *I^2^* statistics, with *I^2^* >50% indicating significant heterogeneity. A fixed-effect model was used for initial analyses, and a random effect model was used if *I^2^* >50%. Sensitivity analyses excluding one study at a time were conducted when the heterogeneity was high, but subgroup analyses and meta-regression were not conducted because of limited literature. Two-sided *P* values were statistically significant if *P*<0.05. Potential publication biases were detected by funnel plots. Given to the limited capacity of funnel plots when pooling a small number of trials, we further preformed Egger’s test to verify the potential publication bias.

## Results

### Search Results

**Figure 1:**
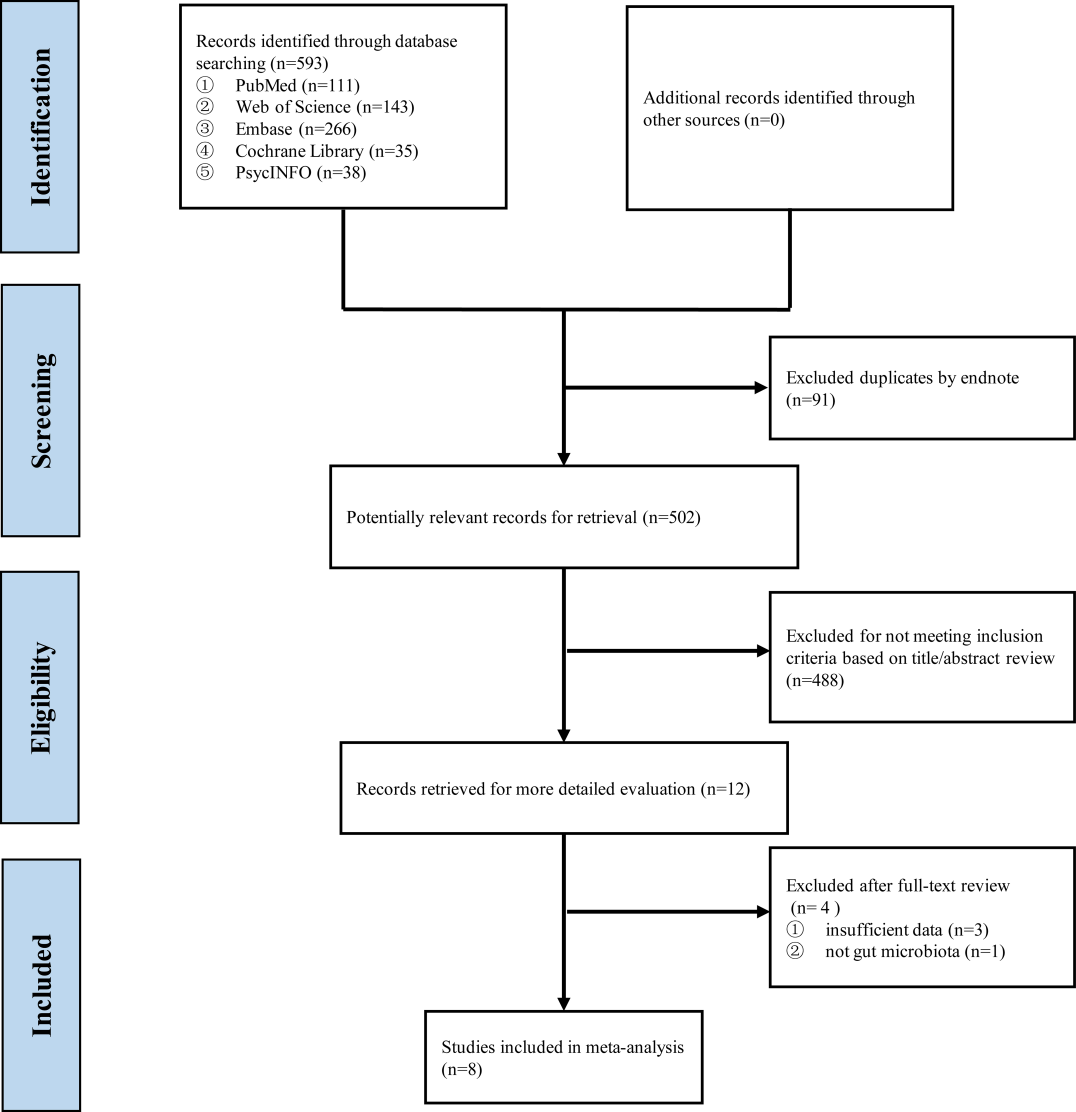
Flow diagram of selected studies.

Up to August 24, 2021, 593 records were found after searching the five databases, and 502 were retained after duplicate manual removal. After screening the title and abstract, 488 studies were removed because of dissatisfaction with the inclusion criteria. After reviewing the full texts of the remaining articles, three were excluded because of a lack of insufficient data, and one was excluded because the data of microbiota is not for gut microbiota. Finally, eight eligible studies were included in this systematic review and meta-analysis (**Figure 1**), and the PRISMA report is presented in **Supplementary Material 2**.

### Study Characteristics

**Table 1** summarizes the characteristics of the eight studies included in the meta-analysis, among which four were conducted in China (including Taiwan) (12–14, 17), two in the Netherlands (10, 15), one in Germany (11) and one in Spain (16). A total of 316 ADHD patients and 359 healthy controls were included in the analysis, and the sample sizes ranged from 14 to 100. Most studies were age- and gender-matched, and there were no significant differences in demographics, except the study by Aarts, in which the HCs had 39 older adults and caused an older mean age (10). For participants, four studies were conducted in children (12–14, 17), one was in children and adolescents (11), two were in adolescents and adults (10, 15), and the last was in adults (16).

**Table 1:** Characteristics of the studies included in the meta-analysis.

Study	Country	N^a^ (ADHD)	Age (years)	Sex (male, %)	N^b^(Control)	Age (years)	Sex (male, %)	Definition of ADHD	Bacteria		Microbiology Assessment	Dietary Assessment	Probiotics Usage Assessment
									Bacteria Identified	Bacteria Altered			
**Aarts et al.** (10)	The Netherlands	19	19.5 (2.5)	68.4%	77	27.1 (14.3) (33 older participants)	53.2%	DSM-IV; Schedule for Affective Disorders and Schizophrenia for School-Age Children	**Phylum:** Firmicutes, Actinobacteria, Bacteroidetes **Order:** Clostridiales **Family:** Rikenellaceae, Porphyromonadaceae **Genus:** *Bifidobacterium*, *Eggerthella*	**Phylum:** Firmicutes↓, Actinobacteria↑ **Genus:** *Bifidobacterium*↑	16S rRNA gene sequencing using 454 pyrosequencing; region: V3-V4; Pipeline analysis: QIIME version 1.2	–	–
**Prehn-Kristensen et al.** (11)	Germany	14	11.9 (2.5)	14 (100%)	17	13.1 (1.7)	17 (100%)	DSM-IV-TR; K-SADS-PL	**Family:** Prevotellaceae, Catabacteriaceae, Porphyromonadaceae, Neisseriaceae, Bacteroidaceae **Genus:** *Bacteroides, Prevotella, Parabacteroides*, *Neisseria*	**Family:** Prevotellaceae↓, Catabacteriaceae↓, Porphyromonadaceae↓, Neisseriaceae↑, Bacteroidaceae↑ **Genus:** *Bacteroides*↑*, Parabacteroides*↓	16S rRNA gene sequencing using Illumina MiSeq; region: V1-V2; Pipeline analysis: Mothur	–	–
**Jiang et al.** (12)	China	51	8.47 (8.47)	38 (74.51%)	32	8.5 (8.47)	22 (68.75%)	DSM-IV; K-SADS-PL	**Phylum:** Firmicutes, Bacteroidetes, Proteobacteria, Actinobacteria **Family**: *Alcaligenaceae*, *Peptostreptococcaceae*, *Moraxellaceae*, *Xanthomonadaceae*, *Peptococcaceae* **Genus**: *Faecalibacterium*, *Lachnoclostridium*, *Dialister*, *Sutterella, Blautia*	**family**: *Alcaligenaceae*↓, *Peptostreptococcaceae*↑, *Moraxellaceae*↑, *Xanthomonadaceae*↑, *Peptococcaceae*↑ **Genus**: *Faecalibacterium*↓, *Lachnoclostridium*↓, *Dialister*↓, *Sutterella*↓, *Blautia*↑	16S rRNA gene sequencing using Illumina MiSeq; region: V3-V4; Pipeline analysis: QIIME version 1.7	Yes	No
**Wang et al.** (13)	Taiwan	30	8.4 (1.7)	23 (76.7%)	30	9.3 (2.2)	18 (60%)	DSM-IV-TR; K-SADS-E	**Phylum:** Bacteroidetes, Firmicutes, Proteobacteria, Fusobacteria, Actinobacteria **Genus:** *Bacteroidetes, Prevotella, Parabacteroides, Phascolarctobacterium, Escherichia Shigella*, *Alistipes, Veillonella, Sutterella, Fusobacteria, Akkermansia*	**Phylum:** Fusobacteria↑ **Genus:** *Fusobacteria*↑	16S rRNA gene sequencing using Illumina Miseq sequences; region: V3-V4; Pipeline analysis: Mothur and QIIME	Yes	–
**Wan et al.** (14)	China	17	8 (7,10)	14 (82.3%)	17	8 (7,9.5)	13 (76.5%)	DSM-V; K-SADS	**Genus:** *Faecalibacterium, Veillonellaceae, Odoribacter, Enterococcus* **Species:** *Faecalibacterium prausnitzii, Lachnospiraceae bacterium, Ruminococcus gnavus, Ruminococcaceae*,*Bacteroides caccae, Odoribacter splanchnicus, Paraprevotella xylaniphila, Veillonella parvula*, *Odoribacteraceae*, *Enterococcaceae*	**Genus:** *Faecalibacterium*↓*, Veillonellaceae*↓*, Odoribacter*↑*, Enterococcus*↑ **Species:** *Faecalibacterium prausnitzii*↓*, Lachnospiraceae bacterium*↓*, Ruminococcus gnavus*↓*, Ruminococcaceae*↓, *Bacteroides caccae*↑*, Odoribacter splanchnicus*↑*, Paraprevotella xylaniphila*↑*, Veillonella parvula*↑, *Odoribacteraceae*↑, *Enterococcaceae*↑	Shotgun metagenomics sequencing using Illumina NovaSeq; Platform: Bowtie2	–	–
**Szopinska-Tokov et al.** (15)	The Netherlands	41	20.2 (4.1)	61%	48	20.4 (3.5)	50%	DSM-IV; K-SADS	**Phylum:** Clostridiales, Firmicutes, Bacteroidetes, Actinobacteria, Proteobacteria, Verrucomicrobia **Genus:** *Coprococcus_2, Prevotella_9, Intestinibacter*	**Genus:** *Coprococcus_2*↓*, Prevotella_9*↓	16S rRNA gene sequencing using Illumina Hiseq sequences; region: V1-V2	–	–
**Richarte et al.** (16)	Spain	100	33 (11)	51%	100	30 (8)	47%	Structured Diagnostic Interview for Adult ADHD (DIVA 2.0), the Structured Clinical Interview for DSM-IV Axis I and II Disorders (SCID-I and SCID-II)	**Phylum:** Bacteroidetes, Firmicutes, Proteobacteria, Actinobacteria, Verrucomicrobia, Candidatus Melainabacteria **Family**: *Eubacteriaceae, Gracilibacteraceae, Lactobacillaceae, Peptostreptococcaceae, Selenomonadaceae, Veillonellaceae, Verrucomicrobiaceae* **Genus**: *Acetivibrio, Alloprevotella, Anaerotaenia, Dialister, Flintibacter, Fucophilus, Gracilibacter, Herbinix, Leclercia, Megamonas, Megasphaera, Odoribacter, Parasutterella, Porphyromonas, Prevotellamassilia, Romboutsia, Vampirovibrio*	**Family**: *Veillonellaceae*↑ **Genus**: *Dialister*↑	16S rRNA gene sequencing using Illumina Miseq sequences region: V3-V4	–	No
**Zhou et al.** (17)	China	44	6.9	–	38	8.6	**-**	DSM-V	**Genus:** *Bifidobacterium, Gemmiger* **Species:** *Shigella, SMB53, uricibacter, Shigella, Bifidobacterium, Collinsella,Ruminococcus, Clostridium, Roseburia*, *Gemmiger, Acinetobacter, Enterococcus, Bacteroides, Streptococcus, Faecalibacterium*	**Genus:** *Bifidobacterium*↓ **Species:** *Shigella*↓*, SMB53*↓*, uricibacter*↓*, Shigella*↓*, Bifidobacterium*↓*, Collinsella*↓*,Ruminococcus*↓*, Clostridium*↓*, Roseburia*↑, *Gemmiger*↑*, Acinetobacter*↑*, Enterococcus*↑*, Bacteroides*↑*, Streptococcus*↑*, Faecalibacterium*↑	16S rRNA gene sequencing using Illumina Miseq sequences; region: V3-V4; Pipeline analysis: QIIME2 version 2020.06	–	–

^a^The number of ADHD patients in each study; ^b^The number of healthy controls in each study ↑: indicating the increase of bacterial taxa; ↓: indicating the decrease of bacterial taxa.

For the clinical diagnosis of ADHD, six studies were assessed according to DSM-IV (10–13, 15, 16), and others followed DSM-5 (14, 17). For the assessment of microbiology, except the one conducted by Wan et al. (14) that used shotgun metagenomics (14), other studies used 16S rRNA gene sequencing (10–13, 15–17). Likewise, there were three pipeline analyses in the included studies, QIIME (10, 12, 17), Mothur (11, 13), and Bowtie2 (14), except for two examinations that did not specify the analyses (15, 16).

We also take care of ADHD medication because it may cause gut microbiota disorders. Three of the included records consisted of medication-naïve participants to compare ADHD patients and HCs (12, 13, 16), one study asked patients to discontinue taking medicine for at least 48 h prior to sampling collection (11), and one explored the effect of medication by removing 19 medicated cases from a regression model (15). For the use of probiotics, two studies asked participants not to receive any probiotics (12, 16). Other studies did not clearly state the usage of probiotics (10, 11, 13–15, 17).

Another aspect to highlight was the preparation of fecal samples. Most studies sequenced each sample of all participants separately. Nevertheless, Zhou et al. (17) made mixed fecal samples of ADHD patients by taking 1.0 g fecal samples from each ADHD child and dissolving them in 10 ml of sterile distilled water (17).

### Assessment of Study Quality

All included studies were assessed for quality using the NOS (**Table 2**). All studies were of high quality and were included in the meta-analysis.

**Table 2:** Newcastle–Ottawa Scale for assessing the quality of the studies included in the meta-analysis.

Author, year	Overall score	Selection				Comparability	Exposure		
		Definition adequate	Representativeness of the cases	Selection of controls	Definition of controls	Comparability of cases and controls	Ascertainment of exposure	Same method of ascertainment for cases and controls	Non-Response rate
**Aarts et al., 2017** (10)	**8**	**1**	**1**	**1**	**1**	**2**	**0**	**1**	**1**
**Prehn-Kristensen et al., 2018** (11)	**9**	**1**	**1**	**1**	**1**	**2**	**1**	**1**	**1**
**Jiang et al., 2018** (12)	**9**	**1**	**1**	**1**	**1**	**2**	**1**	**1**	**1**
**Wang et al., 2020**	**9**	**1**	**1**	**1**	**1**	**2**	**1**	**1**	**1**
**Wan et al., 2020** (14)	**9**	**1**	**1**	**1**	**1**	**2**	**1**	**1**	**1**
**Szopinska-Tokov et al., 2020** (15)	**9**	**1**	**1**	**1**	**1**	**2**	**1**	**1**	**1**
**Richarte et al., 2021** (16)	**9**	**1**	**1**	**1**	**1**	**2**	**1**	**1**	**1**
**Zhou et al., 2021** (17)	**8**	**1**	**1**	**1**	**1**	**2**	**0**	**1**	**1**

### Differences in Diversity Outcomes Between ADHD Patients and HCs

#### Alpha Diversity

**Table 3** presents different kinds of alpha diversity indexes used in the included studies to assess the microbial diversity within the same group, including estimated richness (observed OTUs, observed species, Chao1 index), and indexes presented richness and evenness (Shannon index, Simpson index).

**Table 3:** Summary of diversity assessments in the included studies.

Study	α-diversity	Findings	β-diversity	Findings
Szopinska-Tokov et al. (15)	Observed OTUs Shannon index Phylogenetic index	no difference	weighted UniFrac distances	no difference
Prehn-Kristensen et al. (11)	Observed species Shannon diversity Chao1 index	The ADHD group had lower Shannon diversity than HCs.	Bray–Curtis distance	a significant difference
Wan et al. (14)	Shannon index Chao1 index Simpson index	no difference	–	–
Wang et al. (2020)	Chao1 index Observed OTUs Shannon index	The ADHD group had higher Shannon index and Chao index than HCs. However, the Simpson index was lower in ADHD group.	unweighted and weighted unifrac distances	no difference
Aarts et al. (10)	PD whole tree Chao1 index Observed Species Shannon index	no difference	weighted UniFrac distances	no difference
Jiang et al. (12)	Shannon index Simpson index ACE Chao1 index	no difference	unweighted and weighted UniFrac distances, Bray–Curtis distance	no difference
Richarte et al. (16)	Simpson index Shannon index	no difference	unweighted and weighted UniFrac distances, Bray–Curtis distance	no difference
Zhou et al. (17)	Shannon index Simpson index Pielou’s evenness	The ADHD group had higher indexes than HCs.	weighted UniFrac unweighted UniFrac Jaccard distance Bray–Curtis distance	a significant difference

**Figure 2:**
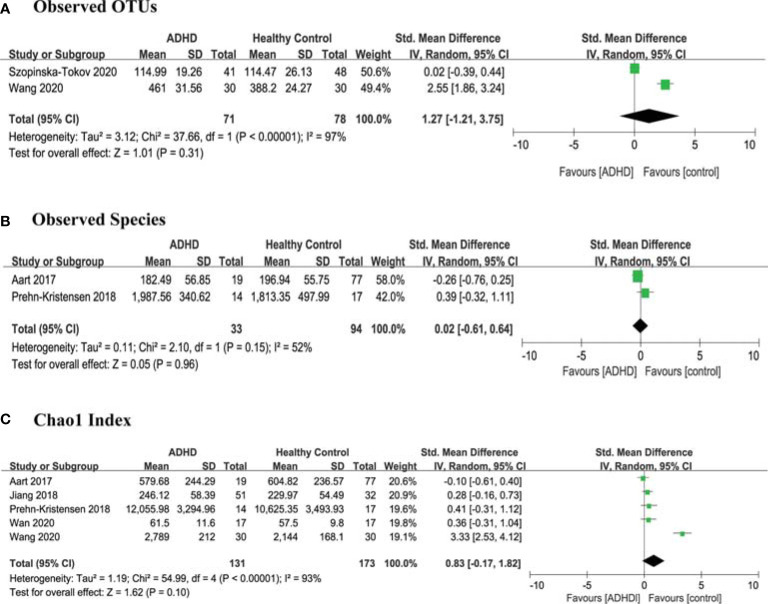
Forest Plots of Alpha Diversity Richness Estimators in the Gut Microbiota of ADHD Compared with HCs. **(A)** Observed OTUs; **(B)** Observed Species; **(C)** Chao1 index. CI, confidence interval; SMD, standardized mean difference.

For richness, 2 studies (13, 15) provided data on observed OTUs in ADHD patients (n=71) vs HCs (n=78), 2 studies (11, 16) provided observed species in ADHD (n=33) vs HCs (n=94), and 5 studies (10–14) provided Chao1 in ADHD (n=131) vs HCs (n=173). There were no significant differences in SMDs of observed OTUs (SMD = 1.27; 95% CI, −1.21 to 3.75; *P* = 0.31; *I^2^* = 97%) (**Figure 2A**), observed species (SMD = 0.02; 95% CI, −0.61 to 0.64; *P* = 0.96; *I^2^* = 52%) (**Figure 2B**) or Chao1 (SMD = 0.83; 95% CI, −0.17 to 1.82; *P* =0.10; *I^2^* = 93%) (**Figure 2C**) indexes.

**Figure 3:**
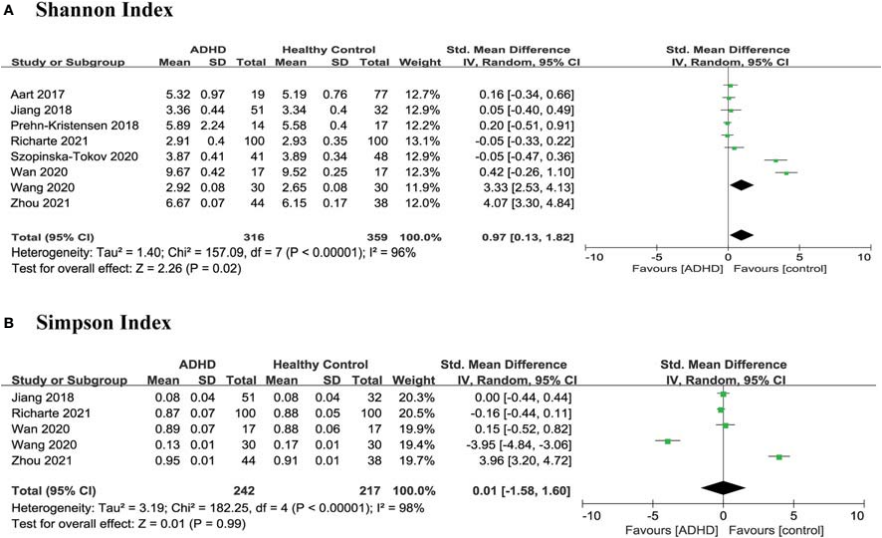
Forest Plots of Alpha Diversity richness and evenness in the Gut Microbiota of ADHD Compared with HCs. **(A)** Shannon index; **(B)** Simpson index. CI, confidence interval; SMD, standardized mean difference.

Regarding richness and evenness, 8 studies (10–17) provided data on the Shannon index in ADHD (n=316) vs HCs (n=359), and 5 studies (12–14, 16, 17) provided the Simpson index in ADHD (n=242) vs HCs (n=217). The estimate demonstrated a higher Shannon index in ADHD patients than in HCs (SMD = 0.97; 95% CI, 0.13 to 1.82; *P* = 0.02; *I^2^* = 96%) (**Figure 3A**) and no significant difference in the Simpson index (SMD =0.01; 95% CI, −1.58 to 1.60; *P* = 0.13; *I^2^* = 96%) (**Figure 3B**).

**Figure 4:**
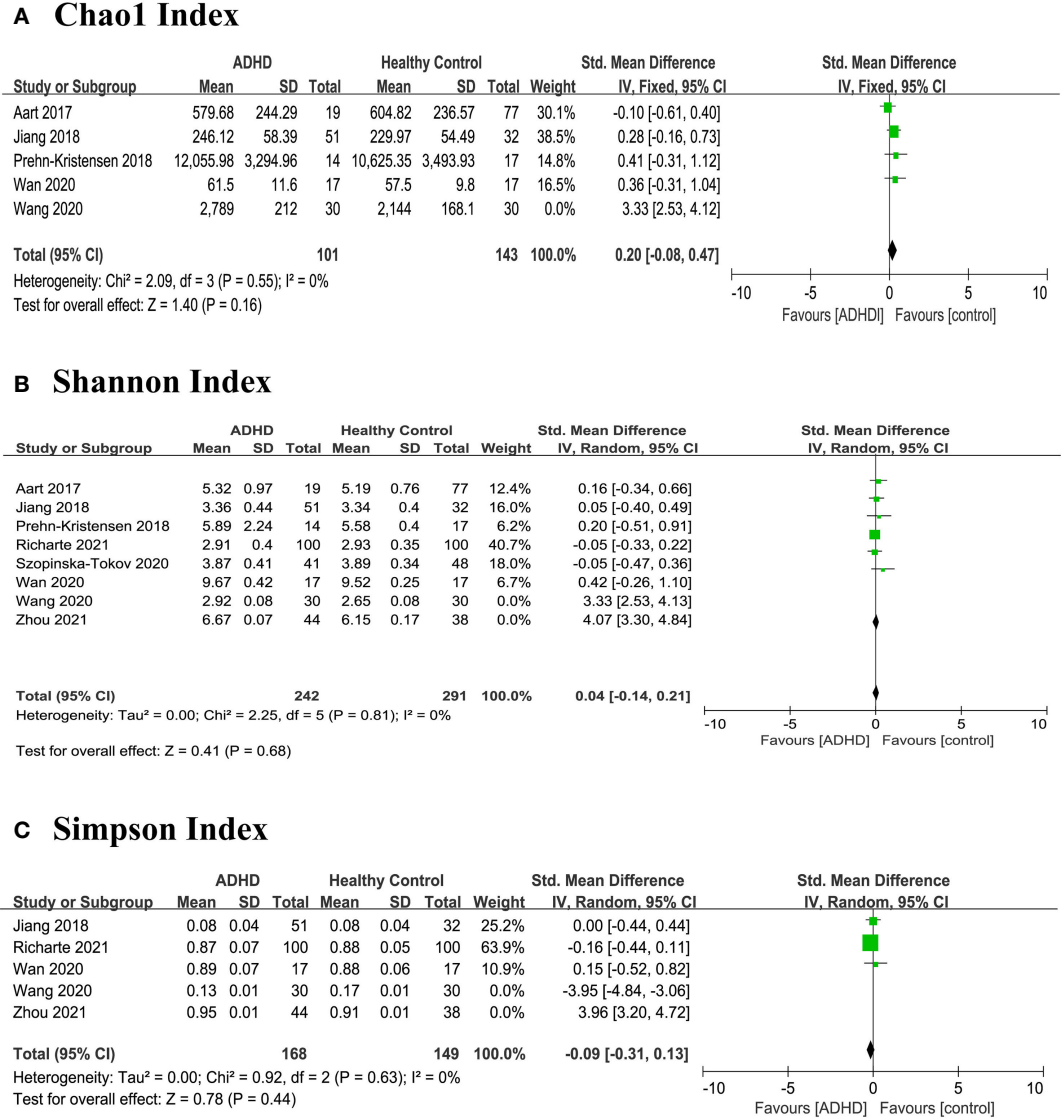
Sensitivity analysis of alpha diversity in the gut microbiota of ADHD compared with HCs after removing heterogeneous studies of Wang 2020 and Zhou 2021 (17). **(A)** Chao1 index; **(B)** Shannon index; **(C)** Simpson index. CI, confidence interval; SMD, standardized mean difference.

In order to explore the high heterogeneity (*I^2^*) of Chao1 index, Shannon index, and Simpson index, we wanted to perform subgroup analyses and meta-regression but gave up because of limited literature. Then, we found that the heterogeneity was skewed by the results from two outlier studies Wang et al. and Zhou et al. (13, 17), and a sensitivity analysis excluded the two studies and produced a homogeneous study population (**Figure 4**). This high heterogeneity could be due to the preparation of fecal samples (17) and pipeline analyses (13) as described in the study characteristics above. However, there were no significant differences between ADHD patients and HCs in any alpha diversity index.

#### Beta Diversity

Seven studies reported four types of beta diversity, and the findings were inconsistent (**Table 3**); five records showed no significant difference between ADHD patients and HCs, while two reported the opposite conclusion. We did not conduct a meta-analysis on beta diversity because of few data.

### Differences in Microbial Taxa Between ADHD Patients and HCs

#### Bacterial Phylum

**Figure 5:**
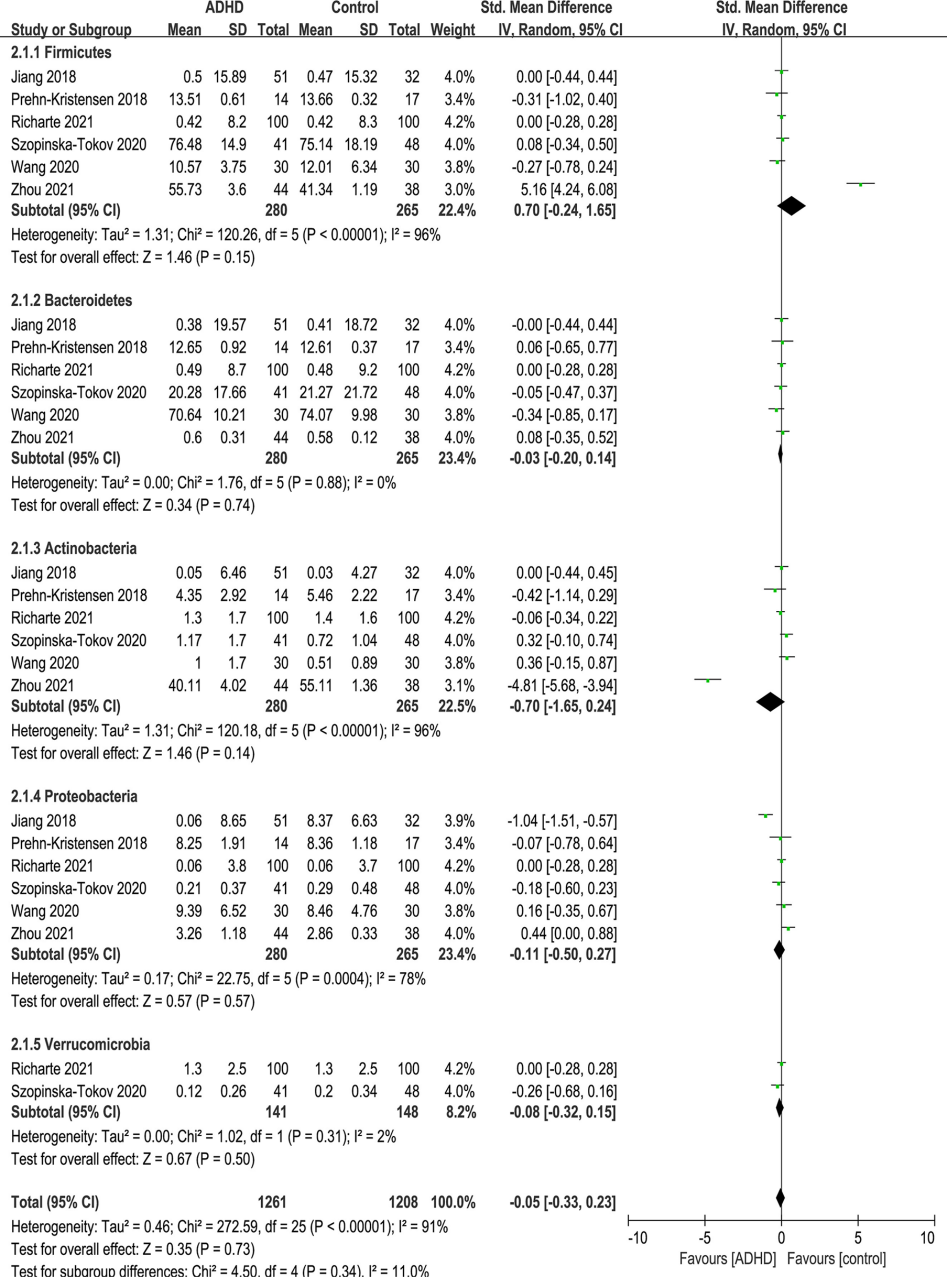
Forest plot of relative abundance of Phylum in the Gut Microbiota of ADHD Compared with HCs. CI, confidence interval; SMD, standardized mean difference.

At the phylum level, five phyla were identified: Firmicutes, Bacteroidetes, Actinobacteria, Proteobacteria and Verrucomicrobia (**Figure 5**). There were no significant differences in phylum.

**Figure 6:**
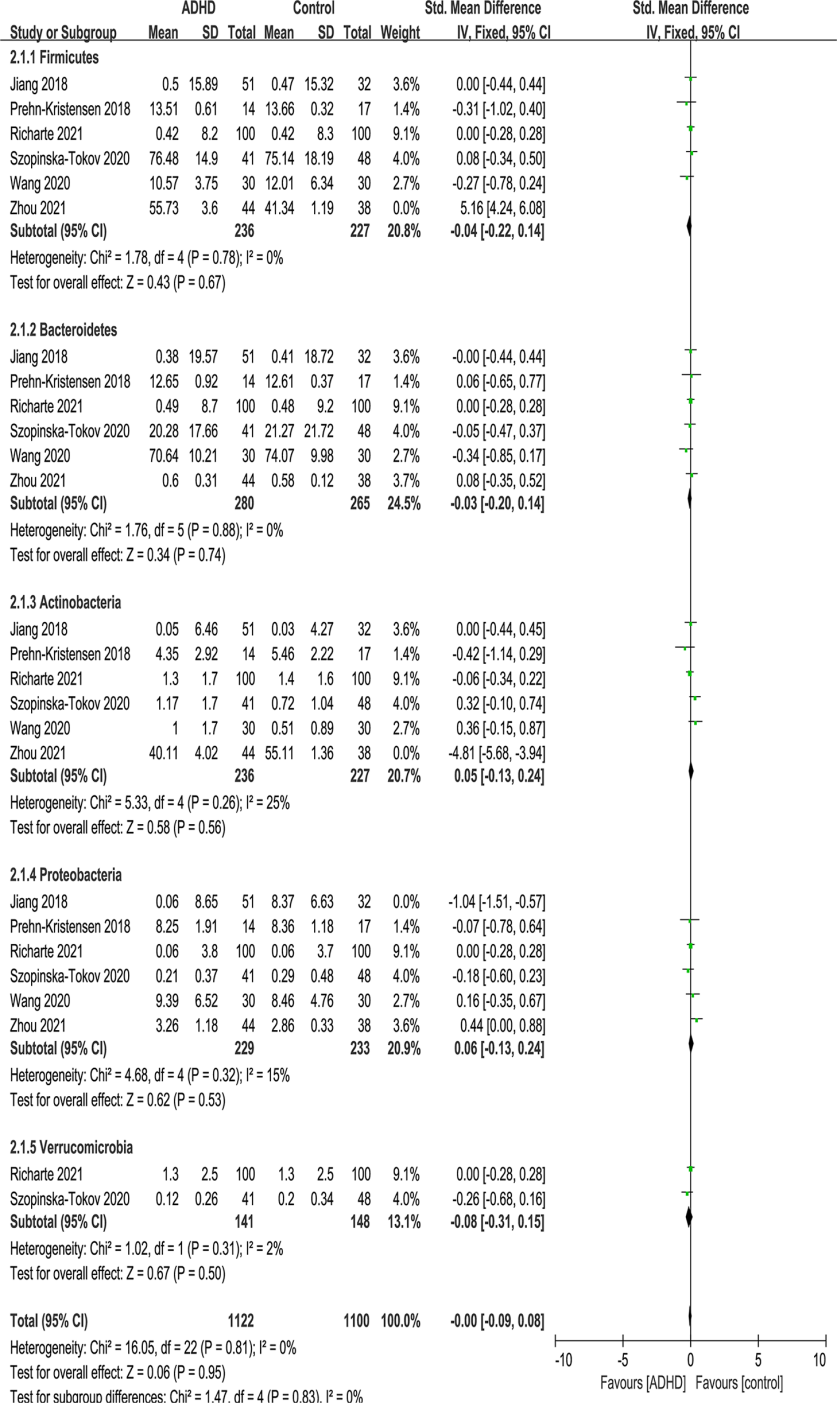
Sensitivity analysis after removing heterogeneous studies of relative abundance of Phylum in the Gut Microbiota of ADHD Compared with HCs. CI, confidence interval; SMD, standardized mean difference.

Because of the high heterogeneity (*I^2^*) of Firmicutes and Actinobacteria, sensitivity analyses excluded the study of Zhou et al. (17) because of the same reason above, and the model was switched from a random-effects to a fixed-effects model, with a modest impact on the result (**Figure 6**).

#### Bacterial Family

**Figure 7:**
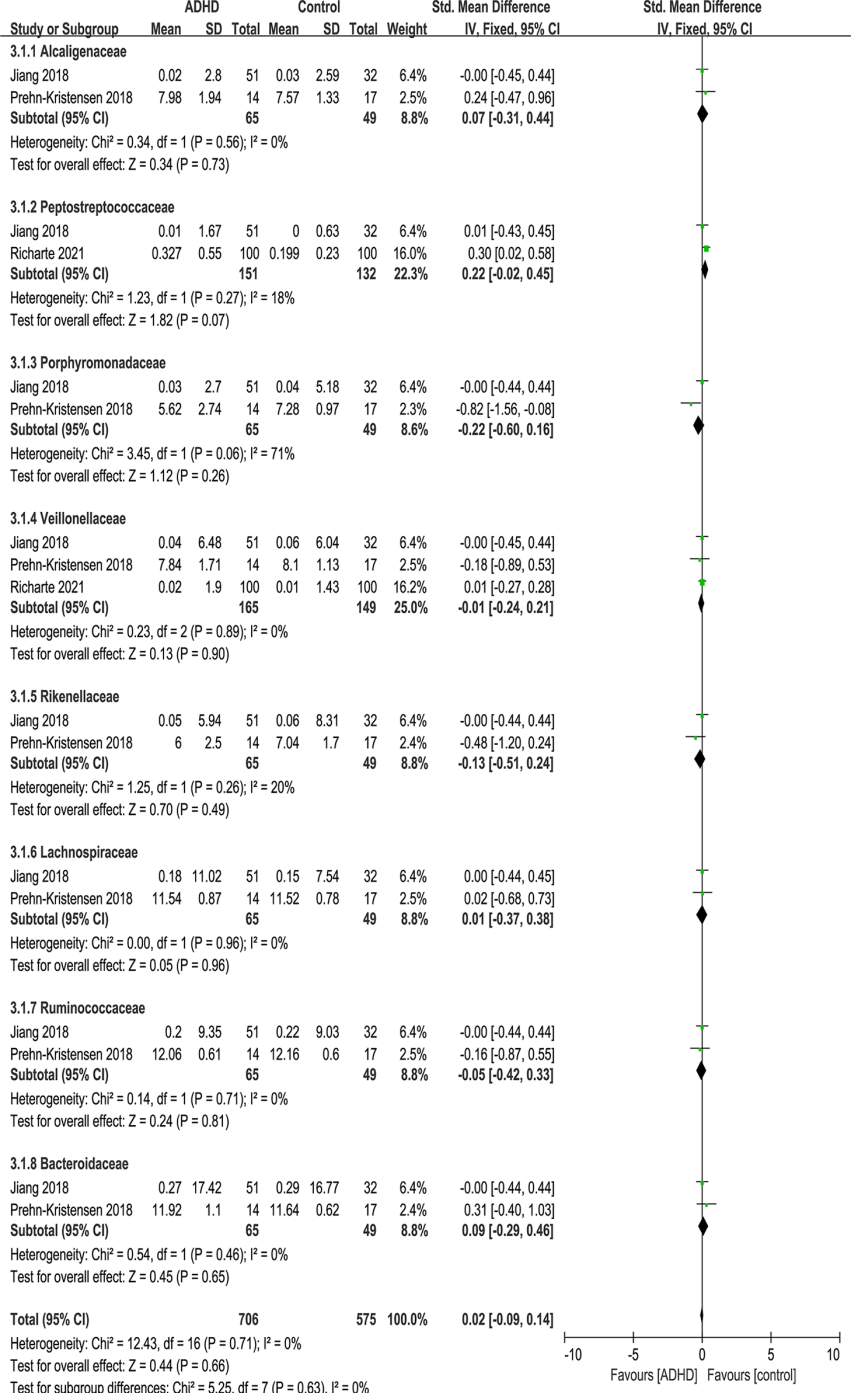
Forest plot of relative abundance of Family in the Gut Microbiota of ADHD Compared with HCs. CI, confidence interval; SMD, standardized mean difference.

At the family level, eight families were identified: Alcaligenaceae, Peptostreptococcaceae, Porphyromonadaceae, Veillonellaceae, Rikenellaceae, Lachnospiraceae, Ruminococcaceae and Bacteroidaceae (**Figure 7**). No significant difference was found in family.

#### Bacterial Genus

**Figure 8:**
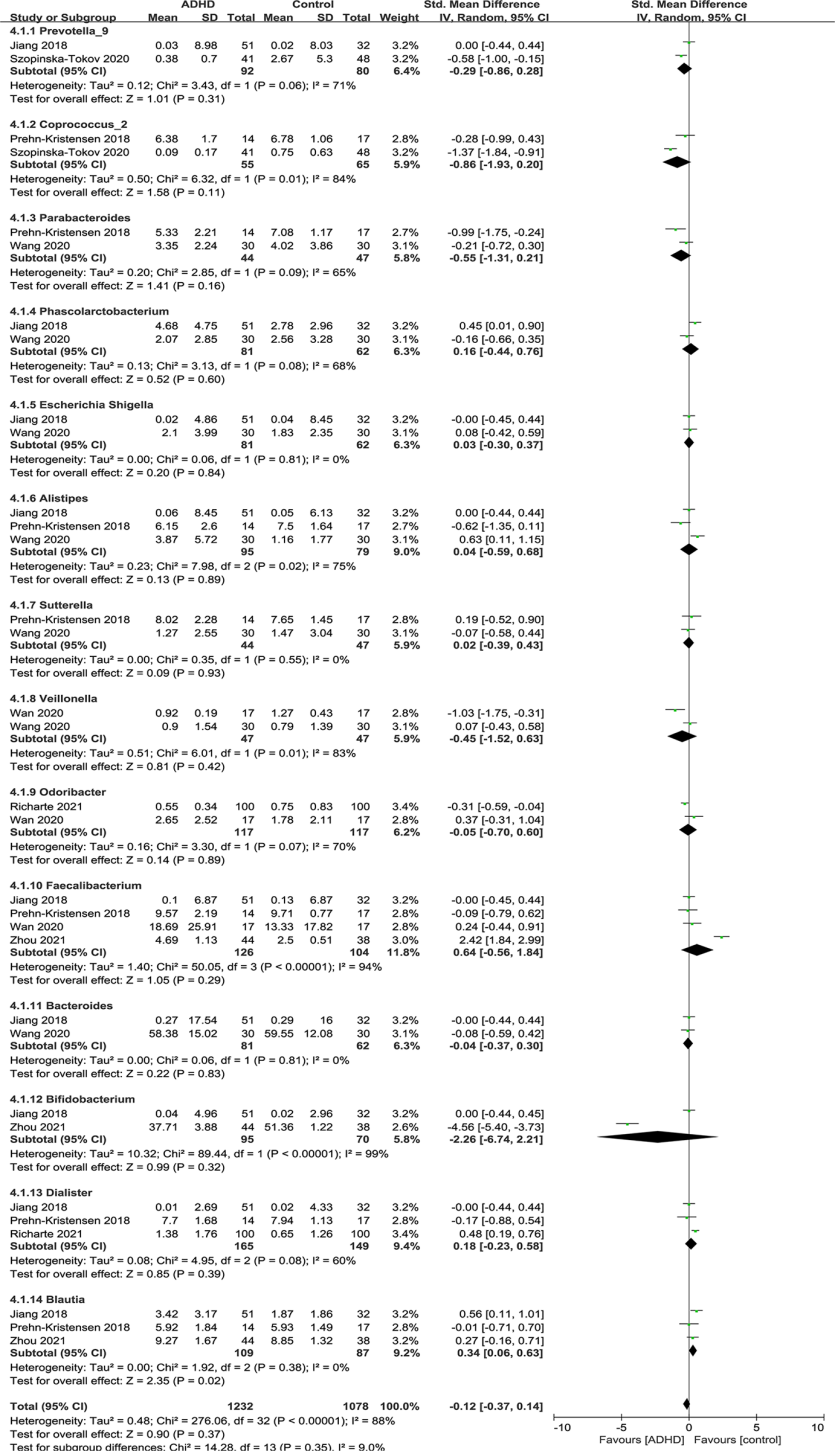
Forest plot of relative abundance of Genus in the Gut Microbiota of ADHD Compared with HCs. CI, confidence interval; SMD, standardized mean difference.

**Figure 8** shows the fourteen genera that were identified: *Prevotella_9* (12, 15), *Coprococcus_2* (11, 15), *Parabacteroides* (11, 13), *Phascolarctobacterium* (12, 13), *Escherichia Shigella* (12, 13), *Alistipes* (11–13), *Sutteralla* (11, 13), *Veillonella* (13, 14), *Odoribacter* (14, 16), *Faecalibacterium* (11, 12, 14, 17), *Bacteroides* (12, 13), *Bifidobacterium* (12, 17), *Dialister* (11, 12, 16) and *Blautia* (11, 12, 17).

**Figure 9:**
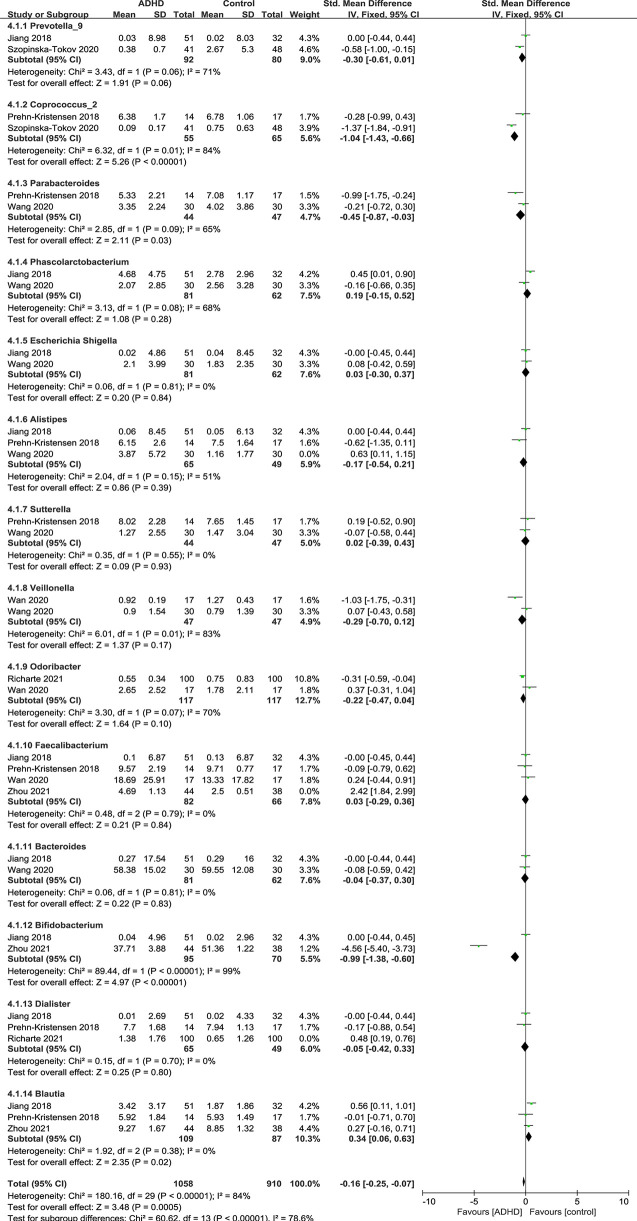
Sensitivity analysis after removing heterogeneous studies of relative abundance of Genus in the Gut Microbiota of ADHD Compared with HCs. CI, confidence interval; SMD, standardized mean difference.

Sensitivity analyses were conducted because of the high heterogeneity of *Alistipes, Faecalibacterium* and *Dialister*, and the model was changed from a random-effects to a fixed-effects model, with a similar result described above (**Figure 9**).

As shown in the forest plot (**Figure 9**), the relative abundance of *Blautia* was significantly higher in ADHD patients than in HCs (SMD = 0.34; 95% CI, 0.06 to 0.63; *P* = 0.02; *I^2^* = 0%). For other genera, no significant difference was found.

**Table 4** summarizes the outcomes of the included studies on microbiota profiles (alpha and beta diversity) and gut microbiota taxa. Different studies did not draw consistent conclusions. For α-diversity, five studies reported nonsignificant differences, but Prehn-Kristensen et al. (11), Wang et al. (13, 18), and Zhou et al. (17) gave different outcomes. Wang et al. (13, 18) and Zhou et al. (17) found a higher Shannon index, but they reached contradictory conclusions on the Simpson index, which may be led by different pipeline analyses of Mothur and QIIME. Prehn-Kristensen et al. (11) disagreed because he found a decrease in the Shannon index. Seven studies addressed β-diversity, with two believed significant differences in all four indexes, while others derived opposite findings. Regarding gut microbiota taxa, different researchers reached different or even contrary conclusions, as shown in **Table 4**.

**Table 4:** Summary of the outcomes of the included studies on microbiota profiles (alpha and beta diversity) and gut microbiota taxa.

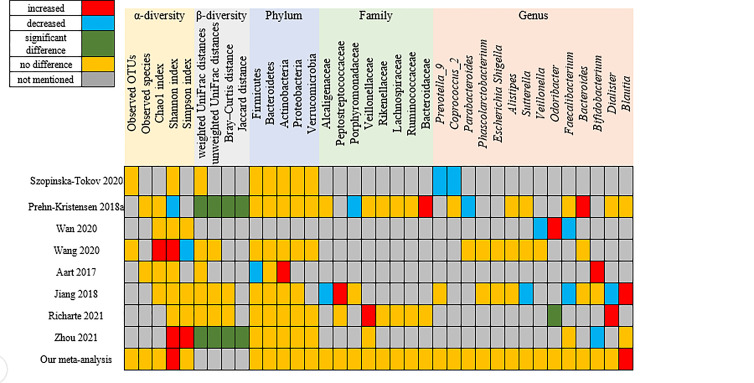

Study references: Szopinska-Tokov et al. (15), Prehn-Kristensen (11), Wan et al. (14), Wang et al. (2020), Aarts et al. (10), Jiang et al. (12), Richarte et al. (16), Zhou et al. (17).

### Publication Bias

Potential publication biases were observed in funnel plots of Chao1 index and Shannon index which were presented in **Supplementary Material 3**. Egger’s test further confirmed the significant bias in Shannon index (*P* = 0.050), but not in Chao1 index (*P* = 0.218).

## Discussion

To our knowledge, this is the first meta-analysis to identify evidence on the dysbiosis of gut microbiota in ADHD. We searched five important databases to accumulate evidence on whether ADHD patients have a different gut microbial composition than healthy controls. A total of eight studies with high quality were included, including 316 ADHD patients and 359 healthy controls. Then, we investigated the diversity and relative abundance of the gut microbiota, more specifically at the 5 phyla, 8 families and 14 genera. Our findings are as follows. First, for the alpha diversity of ADHD patients and HCs, we only found a higher Shannon index in ADHD, but the significance vanished after sensitivity analysis because of high heterogeneity. Second, at the phylum level, no significant difference was found. And at the family level, there was no difference between ADHD and HCs. Finally, at the genus level, *Blautia* was significantly elevated in ADHD patients.

It is worth noting that several systematic reviews (7, 19, 20) summarized differences in gut microbiota between the ADHD group and healthy group but did not draw a final conclusion. They led to a conflicting or even opposite conclusion.

Regarding the alpha diversity of gut microbiota, we found that the Shannon index, which provides information on richness and evenness of gut microbiota, was elevated in ADHD patients, which meant that the within-group diversity was higher in the ADHD group. The result of Shannon index was consistent with reports drawn by Wang et al. (13, 18) and Zhou et al. (17), but we found the heterogeneity was high, and coincidentally, the two studies of Wang et al. (13, 18) and Zhou et al. (17) contributed to it. The possible reasons for this might be the difference in the fecal sampling method of Zhou et al. (17) and pipeline analyses of Wang et al. (13, 18). After sensitivity analysis which excluded the two outlier studies, the difference of Shannon index disappeared. For beta diversity, we did not conduct a meta-analysis due to the inadequate number of studies with available data. Therefore, further studies are needed to explore the association between the diversity of gut microbiota and ADHD.

For specific gut microbiota taxa, we selected bacteria that had two or more studies with sufficient data in the meta-analysis. Our findings that there were no significant differences in bacterial phyla and families were not entirely in tune with previous studies (7, 20). Some studies reported an increased or decreased level of phyla or families, but most studies were in agreement with our study. For the bacterial genus, we found that *Blautia* was significantly higher in ADHD patients, which may serve as a biomarker for ADHD. But there still needs more evidence to verify because of the limited number of studies currently.

*Blautia* belongs to the Lachnospiraceae family, Firmicutes phylum, and contains 20 kinds of species as of now (21). Several recent studies have indicated that *Blautia* is associated with host dysfunctions, such as depression (22, 23), obesity (24, 25), atherosclerosis (26, 27), diabetes (28), and cancer (29), and we now extend these findings to ADHD. This may relate to the functions of physiological of *Blautia*. First, *Blautia* can upregulate T cells (30) in the gut and produce short-chain fatty acids (18) as well as influence the ratio of IFN-γ to IL-4 or TNF-α to IL-4 (31) to achieve anti-inflammatory effects (32). Second, Blautia can produce bacteriocins (33), a kind of secondary metabolite whose function is to prevent the infection of opportunistic pathogens (34). Third, one of the metabolites of *Blautia* is acetic acid, which may modulate other gut microbiota by increasing IgA and changing the capacity of the IgA pool to bind to specific microorganisms (35) and cause a change in gut stability. As inflammation and immunity are substantial etiologies of ADHD, *Blautia* is a possible biomarker of ADHD.

Another point to highlight is that several studies have demonstrated that the use of probiotics or prebiotics may improve ADHD symptoms (19, 36), but we did not conduct an analysis, as most studies included in this meta-analysis did not report on this topic clearly.

In fact, a few limitations should be considered in the meta-analysis. First, the small number of studies and the low to medium sample sizes of each study made the statistical power limited. Other limitations should take into account are geographical location, age, the use of medication, and diet pattern, which may affect outcomes, suggesting that further clinical studies need to be improved to consider these factors. In addition to the reasons described above, a few other factors may also cause high heterogeneity. We did not conduct subgroup analyses of sampling method, sampling time, sequencing, or analysis pipelines because of the limitations of the included literature. However, we performed sensitivity analysis by excluding one or two inappropriate articles when the heterogeneity was high.

## Conclusion

This is the first meta-analysis to assess gut microbiota and ADHD to date. We found a higher Shannon index and *Blautia* in ADHD patients than in HCs, but there were no significant differences at the phylum and family levels. The result for *Blautia* survived the sensitivity analysis. Further clinical studies need to be taken to consider factors such as geographical location, medication use, diet pattern, sequencing and analysis pipelines to validate these results.

## Author Contributions

LY took responsibility for the integrity of the data and the accuracy of the data analysis. Study concept, design and supervision: LY and ZZ. Data extraction, analysis and interpretation: all authors. Drafting of the manuscript: NW and XG. Revision of the manuscript: LY and ZZ. All authors interpreted the results, and approved the final version of this article.

## Funding

This study received funding from National Natural Science Foundation of China (grant numbers: 81873803, 81761128035), Beijing Municipal Science and Technology Commission (Z181100001518005).

## Conflict of Interest

The authors declare that the research was conducted in the absence of any commercial or financial relationships that could be construed as a potential conflict of interest.

## Publisher’s Note

All claims expressed in this article are solely those of the authors and do not necessarily represent those of their affiliated organizations, or those of the publisher, the editors and the reviewers. Any product that may be evaluated in this article, or claim that may be made by its manufacturer, is not guaranteed or endorsed by the publisher.
